# Expanding Brain–Computer Interfaces for Controlling Epilepsy Networks: Novel Thalamic Responsive Neurostimulation in Refractory Epilepsy

**DOI:** 10.3389/fnins.2018.00474

**Published:** 2018-07-31

**Authors:** Abhijeet Gummadavelli, Hitten P. Zaveri, Dennis D. Spencer, Jason L. Gerrard

**Affiliations:** ^1^Department of Neurosurgery, Yale University School of Medicine, Yale University, New Haven, CT, United States; ^2^Department of Neurology, Yale University School of Medicine, Yale University, New Haven, CT, United States; ^3^Department of Neuroscience, Yale University School of Medicine, Yale University, New Haven, CT, United States

**Keywords:** responsive neurostimulation, epilepsy, seizures, centromedian, thalamus, hierarchical networks

## Abstract

Seizures have traditionally been considered hypersynchronous excitatory events and epilepsy has been separated into focal and generalized epilepsy based largely on the spatial distribution of brain regions involved at seizure onset. Epilepsy, however, is increasingly recognized as a complex network disorder that may be distributed and dynamic. Responsive neurostimulation (RNS) is a recent technology that utilizes intracranial electroencephalography (EEG) to detect seizures and delivers stimulation to cortical and subcortical brain structures for seizure control. RNS has particular significance in the clinical treatment of medically refractory epilepsy and brain–computer interfaces in epilepsy. Closed loop RNS represents an important step forward to understand and target nodes in the seizure network. The thalamus is a central network node within several functional networks and regulates input to the cortex; clinically, several thalamic nuclei are safe and feasible targets. We highlight the network theory of epilepsy, potential targets for neuromodulation in epilepsy and the first reported use of RNS as a first generation brain–computer interface to detect and stimulate the centromedian intralaminar thalamic nucleus in a patient with bilateral cortical onset of seizures. We propose that advances in network analysis and neuromodulatory techniques using brain–computer interfaces will significantly improve outcomes in patients with epilepsy. There are numerous avenues of future direction in brain–computer interface devices including multi-modal sensors, flexible electrode arrays, multi-site targeting, and wireless communication.

## Key Concepts

- Epilepsy is a network disorder with potential aberrance in nodes and/or pathways.
- Recent advances in implanted devices with intracranial electroencephalography (EEG), and real-time seizure detection paired with cortical or subcortical stimulation allow for “intelligent” closed loop feedback control.
- Targeting of central nodes such as the thalamus allows for modulation of distributed seizure networks.
- Understanding and characterizing the dynamic and patient-specific epilepsy network requires long-term intracranial EEG studies.
- Future brain–computer interface devices for the treatment of epilepsy are likely to involve a variety of wireless electrode arrays with multi-modal sensing and modulatory capabilities.

## Introduction

### Epilepsy as a Network Disorder

The understanding of seizure generation has traditionally assumed a failure of balance between excitation and inhibition at the seizure onset area. Mixed evidence exists to support uncontrolled excitation at seizure onset (Zaveri et al., 2010; Baud et al., 2018) and this concept has substantially given way to a network theory of epilepsy (Spencer, 2002) where the dominant factor is network aberrance and the role of synchrony in this aberrance (Spencer, 2002; Kramer and Cash, 2012; Richardson, 2012; Blumenfeld, 2014; Dickten et al., 2016; Englot et al., 2016; Smith and Schevon, 2016; Spencer et al., 2018). While an imbalance between excitation and inhibition remains a rational theory it is now considered to play a secondary role and not necessarily the mechanism which governs the behavior of the seizure generating network (SGN).

As indicated in a recent review (Spencer et al., 2018), studies of epilepsy networks in humans have focused on time-series analysis of seizures (Spencer, 2002; Kramer et al., 2008; Schindler et al., 2008; Terry et al., 2012; Varotto et al., 2012; Bialonski and Lehnertz, 2013; Jiruska et al., 2013; Burns et al., 2014; Sritharan and Sarma, 2014; Yaffe et al., 2015; Dickten et al., 2016; Khambhati et al., 2016; Smith and Schevon, 2016; Smith et al., 2016; Geier and Lehnertz, 2017) and to a lesser extent of interictal activity (Towle et al., 1998; Schevon et al., 2007; Zaveri et al., 2009a; Frei et al., 2010; Warren et al., 2010; Negishi et al., 2011; Palmigiano et al., 2012; Varotto et al., 2012; Constable et al., 2013; Lee et al., 2014; Englot et al., 2015, 2016; Nissen et al., 2017; Sinha et al., 2017; Tomlinson et al., 2017). The analysis methods used have included linear and non-linear measures of relationships and models of networks and network dynamics. Studies performed with scalp EEG and intracranial EEG (icEEG) have demonstrated a spatially widespread and profound change in functional connectivity and network measures during seizures. Studies performed with EEG, icEEG, fMRI, and magnetoencephalography (MEG), during the interictal period, have also demonstrated the presence of spatially widespread changes in functional connectivity and network measures (Schevon et al., 2007; Zaveri et al., 2009a; Frei et al., 2010; Warren et al., 2010; Negishi et al., 2011; Palmigiano et al., 2012; Varotto et al., 2012; Constable et al., 2013; Lee et al., 2014; Englot et al., 2015, 2016; Nissen et al., 2017; Sinha et al., 2017; Tomlinson et al., 2017). In fact, observations from some of the interictal studies have been used to predict surgical outcome suggesting that elements of the SGN are persistently active and can be identified in the interictal period (Sinha et al., 2017; Tomlinson et al., 2017). Animal studies (Cleeren et al., 2016; Albright et al., 2017; Wang et al., 2017; Li et al., 2018; Sheybani et al., 2018) have confirmed the involvement of a large-scale network and also suggest that this network evolves through epileptogenesis with both increasing and increasingly widespread manifestation of interictal phenomena such as EEG spikes and high frequency oscillations (HFOs). The human and animal studies together indicate that the SGN is a large-scale network which can be detected in the interictal state and which evolves over time.

**FIGURE 1:**
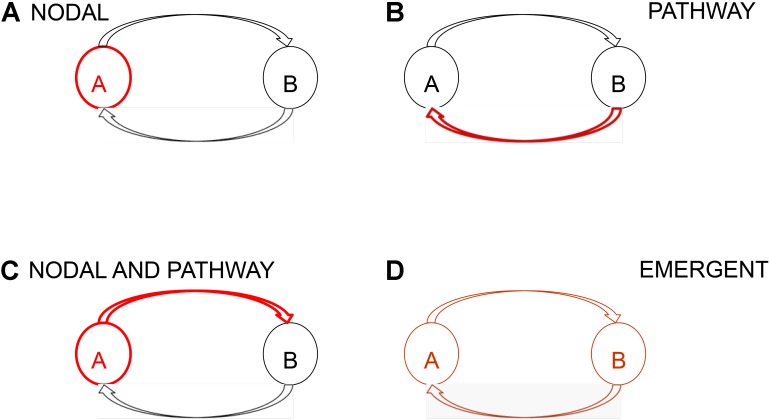
Schematic representation of a network and network aberrance. At its most elemental a network consists of two nodes and two directed pathways, one from each node to the other. Absent aberrant input into a network, network aberrance can result from **(A)** nodal, **(B)** pathway, **(C)** nodal and pathway, and **(D)** emergent aberrance. In emergent aberrance, even though the individual components of the network are not aberrant the resultant network is aberrant because of the structure of the network. Network aberrance may be transient and may manifest only when certain conditions are met.

We consider brain networks to be composed of a functional and anatomically connected set of cortical and subcortical areas (Spencer, 2002). At its most elemental, a network will consist of two nodes and two directed pathways, one from each node to the other (**Figure 1**; Spencer et al., 2018). There are multiple manners in which even a simple network such as this can become aberrant (**Figure 1**). These include the situation where one or more components of the network are aberrant, for example one or both nodes are aberrant; or one or both pathways are aberrant; or that while none of the components are aberrant the network as a whole is aberrant as an emergent property. Which nodes and pathways form an epileptic network, the nature of its aberrance, and whether this network is extant or novel, or whether the network is static or dynamic has not been fully established. It is not known if the SGN precedes the first seizure or forms with it, or whether interictal or ictal activity, or both, inform the network and even strengthen it as suggested by clinical experience and recent reports on the presence of seizure activity patterns during the inter-ictal state (Bower et al., 2015, 2017). The hierarchical structure of the SGN is also not known. For example, are some nodes more important than others? That is, are some nodes central and others peripheral or secondary? Further, are some networks subsumed within others, essentially as sub-networks?

In recent years, we have seen the emergence of open- and closed-loop implantable devices for the control of seizures (Cook et al., 2013; Heck et al., 2014; Sun and Morrell, 2014; Boon et al., 2018; Sun et al., 2018). Closed-loop devices offer a particular strength for the control of seizures which arise from a network in that they can monitor brain activity for an extended period of time, allowing a determination of the areas and times of vulnerability and in this manner allowing a potential assessment of the network dynamics which underlie seizure generation. The network theory of epilepsy, however, poses unique challenges for the control of seizures with a brain–computer interface (BCI). These challenges lie in the spatial scale and dynamics of the network. There is a need to monitor the multiple components of the network, which may or may not be explicitly defined at the time the BCI is established and which may evolve with time. Furthermore, intervention cannot be expected to be uniformly successful if it is based on electrical stimulation because the nodes and pathways of the network may not all respond to electrical stimulation in the same manner.

Here we describe the use of the NeuroPace RNS device for monitoring both neocortical and thalamic activity with the consideration that these are two nodes of a network, with the thalamus being a central node with a broad spatial projection. We have previously demonstrated thalamic involvement in amygdala kindled seizures (Blumenfeld et al., 2007) and demonstrated energetic loss in the anterior nucleus of the thalamus (ANT) ipsilateral to the epileptogenic hippocampus in patients with 7T MR spectroscopy (Pan et al., 2013). Furthermore, we have argued that the lack of an observation of increasing excitation prior to seizures in humans suggests that a cascade of network failures involving the thalamus and cortex leads to a seizure (Zaveri et al., 2010). Some groups have observed variable involvement of the thalamus in seizures (Guye et al., 2006), while others have observed more consistent involvement of the thalamus (Osorio et al., 2015). This discrepancy could be related to which parts of the cortex and thalamus are monitored as it can be assumed that both should lie on the same circuit if ictal involvement is to be observed. It is possible, also that the ictal involvement may take different forms in different parts of the same network because each node of a network will not necessarily express a seizure in the same manner.

### Failure of Traditional Surgical Therapies

Surgical resection is one of the most rigorous methods in which the concept of the seizure network can be tested. Clinical trials show that resection of a suspected seizure onset zone for patients with localizable medically refractory epilepsy, especially temporal lobe epilepsy is superior to medical therapy (Wiebe et al., 2001; West et al., 2015). However, we must account for surgical failures, which have often been described as a failure to remove the entire seizure onset zone, but may be due to additional epileptogenic nodes within an aberrant network capable of seizure initiation. As reported in a recent review (Spencer et al., 2018) long term seizure freedom is achieved in 30% of frontal lobe epilepsy patients and 50–60% of temporal lobe epilepsy patients. Furthermore, the seizure-free rate drops significantly in the epilepsies outside of the medial temporal lobe, where functional network connectivity is less well studied (Bell et al., 2017). A recent analysis at Yale of long-term seizure control (over 10 years) looked at patients whose ictal onset was identified in the medial temporal lobe by intracranial electrode study and underwent anterior mesial temporal resection. The data showed that the patients most likely to fail after surgical resection had rapid electrographic propagation (within 10 s) from the seizure onset zone to a non-contiguous node (defined as at least 2 cm away) during their icEEG study (Andrews et al., 2017). This indicates that certain properties of the SGN, including functional connectivity may predict response to the current surgical treatments. Understanding the characteristics of the seizure network is important if we intend to modulate seizure initiation, propagation, and frequency. A notable and convergent theme of patients that require neuromodulatory treatment due to failure of traditional treatments (AEDs, lobar and sublobar resection, lesionectomy) is a distributed seizure network with rapid propagation. Furthermore, a longer duration of epilepsy has been reported to predict surgical failure, suggesting the SGN has evolved and strengthened over a period of time. We propose that recent developments using patient-specific targeting of the SGN, based on known epileptogenic circuits, combined with responsive neurostimulation (RNS) and other technologies will improve seizure control outcomes in refractory patients. The development of next generation BCIs with flexible high-density sensors capable of multi-modal sensing, network analysis, and wireless communication is essential in this endeavor.

## Neuromodulation of the Seizure Network

The increasing recognition that epilepsy is a network disorder is gradually leading to new diagnostic and therapeutic approaches. This avenue led to the first FDA approved, closed-loop neuromodulatory device, RNS (NeuroPace^TM^) for the treatment of refractory epilepsy in patients with two or fewer regions of onset. For decades, the surgical treatment of epilepsy has been dominated by a localized versus generalized concept of epilepsy. Although the resulting quest for a resectable focus and lobectomies has greatly improved the lives and outcomes of many patients, it is becoming increasingly clear that a new paradigm is needed.

The network theory in epilepsy recognizes that even localized epilepsy has distributed connectivity and functional impact on widespread brain activity. In fact, network studies are increasingly common in the study of several neurological and psychological disease states and it has been suggested that overlapping networks may explain well known co-morbidities such as depression in epilepsy (Spencer et al., 2018). Therefore there has been an expansion of techniques for measuring global brain activities (i.e., functional imaging) combined with the application of modern mathematical network theory. Graph theory, for example, provides a flexible representation of real-world networks, which provides a framework for examining the topology, and the local and global organization of brain networks (Bullmore and Sporns, 2009, 2012; van den Heuvel et al., 2010).

Understanding the SGN of an individual patient presents the opportunity for neuromodulatory interventions within the nodes and/or pathways of the recognized network. Neuromodulation can involve chronic open-loop stimulation or responsive (closed-loop) stimulation devices and can involve a variety of targets including seizure onset zones and major nodes within the identified network. For many of the identified epilepsy networks, the thalamus is a major node and provides an opportunity to modulate the network. The role of thalamo-cortical networks in generalized epilepsy is well established. Many of the SGNs within the focal epilepsies also involve the thalamus. For example, limbic network nodes and pathways that are commonly utilized for neurostimulation include the hippocampus proper, fornix, and the ANT, all of which have been targeted with deep brain stimulation (DBS). The nodes within the other commonly recognized epilepsy networks (i.e., frontal-parietal, occipital-temporal) are less well established, but there are other thalamic nuclei that have been targeted in DBS for epilepsy. The centromedian (CM) and centrolateral (CL) intralaminar nuclei of the thalamus have widespread connectivity with association cortices; DBS targeting the CM has been reported in several small studies as reviewed below. The CM nucleus is also known to play a role in wakefulness in addition to widespread cortical excitability.

### Hippocampus

Medial temporal lobe epilepsy (MTLE) is the most common form of medically refractory epilepsy and the hippocampus is an appealing target for neuromodulation. Although AMTR is a good surgical option for most patients with MTLE, hippocampal resection or ablation may be contraindicated, for example in cases with dominant MTLE and preserved verbal memory or in cases of bitemporal onset. It should be noted that the hippocampus is larger than most other neuromodulation targets and therefore significant variability in targeting and stimulation may exist. The first systematic human studies were reported by Velasco A.L. et al. (2000) who reported on 16 patients with short term and longer term stimulation of the hippocampus. More recently this group reported an 18 month follow-up on a group of nine patients, four of whom were seizure free. All of the seizure free patients showed early and dramatic responses to hippocampal stimulation (Velasco et al., 2007).

Several other groups have reported series of patients with hippocampal DBS. Boon et al. (2007) reported on acute and long-term hippocampal stimulation in 12 patients. Outcomes from the last 6 months of follow-up (mean total follow-up 33 months, range 15–52) showed one patient was seizure free, six patients with >50% reduction in seizures, and two patients had 30–49% decrease in seizures (Boon et al., 2007). A small series of four patients with left MTLE and MRI evidence of hippocampal sclerosis reported by the University of Western Ontario group had more variable results. In a blinded 6 month period with randomized, crossover design including three consecutive 2 month periods randomized to on-off versus off-on, none of these patients were seizure-free and the median seizure frequency reduction of 15% in the 3 months on versus off was not statistically significant (Tellez-Zenteno et al., 2006). Boex et al. (2011) reported on 8 temporal lobe epilepsy patients unable to undergo AMTL. Two patients became seizure free (25%) and four patients had >50% reduction in seizures (Boex et al., 2011). Although some of these reports and others suggest good therapeutic effect, there have not been any level 1 evidence studies performed on hippocampal DBS for epilepsy to date.

### Anterior Nucleus of the Thalamus (ANT)

**FIGURE 2:**
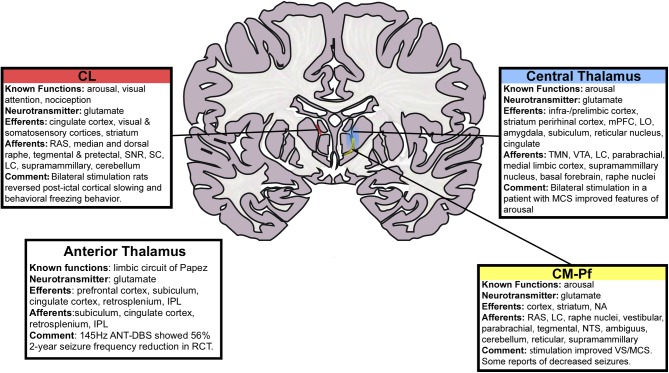
Schematic of thalamic nuclei for stimulation in epilepsy. Deep brain stimulation of the centromedian (CM) and anterior thalamic nuclei (ANT) have been performed previously. The centromedian nucleus has distributed connections with sensori-motor association cortex while the anterior thalamic nucleus is a node in the limbic circuitry. We sought to use a RNS device to control seizures through responsive stimulation of both a cortical and a thalamic node. The cortical node was in the parietal lobe and the thalamic node was in the centromedian nucleus. That is, with reference to the schematic in **Figure 1**, node A was in the parietal lobe and node B was the centromedian nucleus of the thalamus. CL, central lateral; RAS, reticular activating system; SNR, substantia nigra pars reticulate; SC, superior colliculus; LC, locus coeruleus; IPL, inferior parietal lobule; ANT, anterior nucleus of thalamus; DBS, deep brain stimulation; mPFC, medial prefrontal cortex; LO, lateral orbitofrontal cortex; TMN, tuberomammillary nucleus; VTA, ventral tegmental area; MCS, minimally conscious state; CM-Pf, centromedian–parafasciular nucleus; NA, nucleus accumbens; NTS, nucleus tractus solitarius; VS, vegetative state. This figure is adapted with permission from Gummadavelli et al. (2015a).

The ANT is a relatively large structure in the anterior dorsal portion of the thalamus and consists of several distinct subnuclei, some of which have extensive frontal and temporal cortical projections while others are key nodes in the limbic circuit of Papez (see **Figure 2**). Thus, the ANT has been an attractive target for modulating the limbic seizure network, frontal-temporal networks and overall thalamocortical excitation. The ANT receives input from hippocampal subiculum (via the fornix and mamillothalamic tract), anterior cingulate, posterior cingulate, retrosplenium and inferior parietal lobule and generates reciprocal output to many of the same brain regions. Sub-nuclei within ANT may play a role in differential functions: the anteromedial nucleus, in relay of emotional information to the prefrontal cortex; anterodorsal nucleus, in alertness in episodic memory and spatial navigation; anteroventral nucleus, in spatial cognition.

Early studies of both cats and non-human primates suggested an effective reduction in seizure frequency and duration due to lesions in the ANT (Mullan et al., 1967; Kusske et al., 1972). Several small, open label studies of DBS in the ANT during the 2000s showed promising decreases in seizure frequency (30–90%) with some implantation and carryover effects and have been extensively reviewed, as by Laxpati et al. (2014). These promising results were part of the foundation for the Stimulation of the Anterior Nucleus of the Thalamus for Epilepsy trial (SANTE; NCT00101933, Medtronic, Minneapolis, MN, United States). The SANTE trial was a multicenter, randomized, double-blinded trial of bilateral DBS to the ANT for a variety of localization-related epilepsies (Fisher et al., 2010). The 110 participants were randomized at 1 month post-op to receive 3 months of stimulation at 5V (pulse width 90 μs, frequency 145 Hz) alternating at 1 min on and 5 min off or 3 months of sham stimulation. Although there were some important statistical issues in the study and there was a considerable effect of implant (both the stimulation and sham stimulation groups experienced a median reduction in seizures of 21**–**22% at 1 month post-op) there was a significant difference between the treatment groups in the third month of the blinded period (median seizure reduction 40.4% in the stimulation group vs. 14.5% in the sham stimulation group, *p* = 0.0017). The open-label period that followed continued to suggest effectiveness. In fact, the long-term outcomes showed continued improvement with the median seizure frequency decreased by 56% at year 2 and 69% at year 5 (Salanova et al., 2015). These results led to the European CE Mark Approval for DBS Therapy for Refractory Epilepsy and ANT DBS is increasingly utilized for the treatment of refractory epilepsy in Europe. Recently, the FDA also approved ANT DBS for the treatment of partial onset epilepsy in the United States.

### Centromedian Nucleus

The CM nucleus of the thalamus has widespread projections to the cortex with a central role in cortical excitability and wakefulness (**Figure 2**). The circuitry has been suggested to play a role in epilepsy networks during seizure initiation, seizure propagation and also potentially in the loss of consciousness during seizures. In addition, CM has efferents to the striatum, making it well connected to modulate the cortico-striato-thalamic circuitry as well. Velasco et al. (1987) reported on their first five cases of CM DBS for the treatment of epilepsy. This was followed by larger series in 2000 and 2006 (Velasco M. et al., 2000; Velasco et al., 2006). Seizure frequency was measured during a 1 month baseline period and monthly for 18 months. They reported an improvement in the seizure frequency and severity, with the most clearly positive results seen in 13 patients with Lennox-Gastaut syndrome (Velasco et al., 2006). Two of these patients became seizure free and eight patients had >50% reduction in seizures (Velasco et al., 2006). As consistently reported with neuromodulatory therapies, they note an improvement in seizure frequency over time.

Fisher et al. (1992) attempted to study CM DBS in a controlled and blinded fashion though with only six patients. There was a 30% decrease in seizures during the stimulation versus an 8% decrease in the sham group. These results were not statistically significant given the small number of patients (Fisher et al., 1992). The low number of patients enrolled make this study difficult to evaluate. Andrade et al. (2006) reported on two patients with CM DBS where one of the two patients experienced a >50% reduction in seizures. In 2013, a larger study in Europe reported on 11 patients with CM electrodes at two centers (London and Madrid) (Valentin et al., 2013). The patients underwent single-blinded treatment with 3 months of sham stimulation and 3 months of therapeutic stimulation. This was followed by 6 months of open-label stimulation which was continued in patients for whom the therapy was beneficial. Six of the patients had generalized epilepsy and all of these patients showed >50% reduction in seizures during the blinded phase and 5/6 maintained this benefit for the long term. The other five patients had frontal lobe epilepsy and only one of these five patients had >50% reduction in seizures during the blinded phase. In the long term, open label stimulation period 3/5 patients achieved >50% reduction in seizure frequency and the other two patients did not report any benefit (Valentin et al., 2013).

At Yale, Blumenfeld (2012) have extensively studied the mechanisms which cause loss of consciousness during seizure. They have developed the *network inhibition hypothesis*, which describes the inhibitory effects on the ascending arousal system caused by focal seizures (Blumenfeld, 2012). The centromedian (CM) and central lateral (CL) intralaminar nuclei of the thalamus are thought to play a role in the arousal system (**Figure 2**). Studies in rodent models of temporal lobe epilepsy have suggested that neurostimulation in the intralaminar thalamus is capable of reversing the commonly noted post-ictal cortical slow waves (Gummadavelli et al., 2015b) and may participate in the reversal of widespread cortical slow waves seen during temporal seizures. Neuromodulation of intralaminar thalamic nuclei and other nuclei of the ascending arousal system during seizures to restore consciousness remains under active investigation at our center (Gummadavelli et al., 2015a,b; Motelow et al., 2015; Kundishora et al., 2017).

Other DBS targets for neurostimulation for the treatment of epilepsy have included the subthalamic nucleus (STN), caudate, and cerebellum. The results for these targets are variable. The cerebellum was the first widely utilized target for neurostimulation in the treatment of epilepsy. Initial reports by Cooper et al. (1973, 1976) suggested efficacy, however, subsequent controlled studies did not show improvement in any of the patients studied (Van Buren et al., 1978; Wright et al., 1984).

### Responsive Neurostimulation – Toward Brain–Computer Interface Systems

One of the limitations to DBS and vagus nerve stimulation (VNS) is the “open loop” nature of the stimulation. That is to say that the stimulation is either on continuously or cycles in a loop of on-and-off stimulation times regardless of the patient’s ictal state. Morrell and RNS System in Epilepsy Study Group (2011) reported the results of the first pivotal trial utilizing RNS in the first closed loop implantable neurostimulation device (The RNS^®^ System, NeuroPace, Mountain View, CA, United States). The RNS^®^ System provides cortical or subcortical stimulation via a cranially implanted programmable neurostimulator that is connected to one or two recording/stimulating electrode arrays consisting of four contacts in either strip or depth electrode configurations. The design of this trial was to deliver stimulation to the region of seizure onset in response to epileptiform electrographic events. Two hundred and forty subjects were enrolled in the RNS System Pivotal Trial, which was a randomized, double-blind, multicenter, sham-stimulation controlled study. Anti-epileptic medications were kept stable throughout the blinded period of the trial. At the end of the blinded period there was a significant difference in seizure frequency between the sham and treatment groups, with a 37.9% median improvement in the treatment group and a 17.3% median improvement in the sham group (Morrell and RNS System in Epilepsy Study Group, 2011). Both groups showed a decline in seizure frequency following surgical implantation, but the sham group trended back toward baseline, while the treatment group continued to improve. In the following open-label period and even more so in the long-term follow-up there was continued improvement in seizure frequency with the median reduction of 44% at 1 year and 53% at 2 years (Bergey et al., 2015).

Thus, the RNS System represents the first FDA approved “intelligent” or closed-loop neurostimulation device (Heck et al., 2014; Sun and Morrell, 2014; Boon et al., 2018; Sun et al., 2018). It has been increasingly utilized across the United States but is not approved outside the United States. The programmable neurostimulator records continuously on four bipolar channels, from the two electrode arrays that are placed within the seizure onset zone(s). The RNS device supports three main functions as part of its closed loop functionality, monitoring, pattern detection, and electrical stimulation. The device can save a few minutes of data and these data are downloaded off the device onto a provided patient laptop and then into a secure cloud-based storage system.

The device continuously samples the EEG data and uses a variety of feature detectors to detect epileptiform activity within the EEG. The activity detected at two of the four bipolar channels can be subjected to a pattern detector composed of three features. The features are line length (a measure akin to Teager energy; Kaiser, 1990; Zaveri et al., 2009b), band pass filter (e.g., a gamma activity band pass filter), and area under the curve (AUC). The detections can be used to trigger the delivery of therapy which consists of the delivery of electrical charge between electrode contacts or between an electrode contact and the device body. The RNS device has considerable flexibility in the manner in which the feature detectors can be defined and used to trigger stimulation. If necessary the two bipolar channels can work independently of each other. Other functions supported by the device include documentation of events such as detections, “long-episodes,” scheduled ECoG recordings and delivery of therapy. When the detector is triggered, the RNS device delivers a burst of electrical stimulation through the specified programmed electrode contacts. If the RNS detects ongoing seizure activity another burst of stimulation is delivered for up to five total stimulation events (labeled a “long detection”).

The BCI is defined as a technology that allows communication between a human or animal brain and an external technology and often includes the capability of two-way communication. The term BCI can refer to an interface that takes signals from the brain to an external piece of hardware, or a technology that sends signals to the brain or by the strictest of definitions a device and interface that provide for both of the above lines of communication. The RNS system records ECoG or icEEG signals from cortical or subcortical structures, measures the data and responds to epileptiform activity with neurostimulation and thus by many assessments meets the criteria for a BCI. The initial experience with this BCI has been tremendously successful and is already producing the preliminary data and concepts for next generation BCI systems in epilepsy. The availability of long term icEEG data from epilepsy patients alone has provided remarkable data, a new data source that the senior author has referred to as the ECoG revolution. These long-term data have provided immense amounts of information regarding the patient’s epilepsy and have already been utilized for a variety of patient specific therapies such as resection based on long term recordings, the ability to recognize effective versus non-effective medications within a week of starting the new medication (Mercier et al., 2017), and the recognition of slow cycles in seizure activity (Baud et al., 2018). In addition, the RNS provides reliable, objective data regarding the frequency of epileptiform and potential seizure activity from icEEG in the real-world setting.

As the understanding of the neural networks in epilepsy improves and the focus of intracranial studies in patients with refractory epilepsy shifts from the quest to identify a focus to the identification of the network(s) involved in a patient’s epilepsy, it is natural to utilize the RNS system in an effort to modify the activity of the epilepsy network. Thus, rather than simply recording from and stimulating at the proposed seizure onset zone(s), several groups, including ours, have begun to utilize the recordings from a key node within the epileptic network to trigger neurostimulation at another critical node. Until recently, the loss of balance between excitation and inhibition, leading to runaway excitation was thought to underlie seizures. More recently, it has been recognized that neuronal firing rates may not change overall during seizures and it is the aberrant relationship across structures or regions that produce seizures, perhaps via hypersynchrony. Thus, modulating a network in a way that disrupts this aberrant relationship could be therapeutic. Several groups have already recognized the potential to utilize the BCI of the RNS in network modulation, with several reporting the use of depth electrode(s) placed within the ANT to treat refractory epilepsy.

Here we report the first use of the RNS system to monitor and modulate the CM nucleus of the human thalamus and the first ambulatory CM recordings. In the following section we provide the work-up, evaluation and intracranial study of a young patient with refractory epilepsy, including the comprehensive pre-surgical evaluation and intracranial study of the seizure network, seizure onset and propagation. In this evaluation we determined that the patient did not have any resective options and the most likely key node for modulating the proposed seizure network(s) was the CM nucleus. Following epilepsy surgery board review and approval from the VA, the patient underwent the implant of the RNS with a depth electrode array placed within the CM nucleus as well as two strip electrodes placed over the onset region within the posterior parietal lobe. Retrospective review of this case and patient data was approved by IRB.

## Case Presentation: Responsive Centromedian Thalamic Neurostimulation in a Patient

The patient is a 30 year-old right-handed male with bilateral malformations of cortical development (MCD) in right frontal and bilateral inferior temporal periventricular nodules causing medically refractory localization-related epilepsy. He also suffered psychiatric comorbidities of anxiety, panic disorder, and major depressive disorder (MDD). The semiology of his focal unaware seizures were not well lateralized on scalp EEG and are characterized by loss of contact, bizarre behavior, non-sensical speech, or strange vocalizations, lasting 30 s to few minutes, with few minutes of post-ictal lethargy. His seizures began at the age of 16 and he rarely has secondary generalization with his seizures. He occasionally experiences episodes of slowed thinking, dizziness, and heart-racing but this was not reliable for electrographic seizure during scalp EEG recordings and may or may not be a true aura.

Pre-operatively, he had daily seizures with a frequency of 3–6 seizures per day, often occurring in clusters. He was started on topiramate and switched to levetiracetam, lamotrigine, clobazam, clonazepam, lacosamide, and vigabatrin (enrolled in a clinical trial). He underwent treatment with and failed a total of six AEDs prior to surgical consideration. Pre-operative scalp electroencephalography showed bilateral high-frequency seizure discharges, right greater than left in the posterior and temporal regions (max T3, T4). Video-EEG showed 3–12 s high voltage high frequency inter-ictal discharges during sleep, every 10–15 s. He had several recorded seizures with blank stares, no automatisms, lasting 10–31 s with diffuse high-frequency high-voltage poly-sharp rhythmic discharges some appearing to start on the right and some on the left, all localizing posteriorly, suggesting occipital lobe. Functional MRI showed left language dominance. FDG-PET showed decreased uptake in the right medial temporal lobe cortical areas adjacent to the nodules. Neuropsychological testing showed bilateral impairment with prominent difficulties in visuospatial reasoning and integration. In total his work-up was not well lateralizing but suggested involvement of the occipital, parietal, and temporal regions.

The patient underwent a stereotactic depth electrode placement with the robotic-assistance (ROSA, MedTech; Montpellier, France). Multiple depth electrodes were placed in the bilateral parietal, temporal, occipital, and frontal lobes with several electrodes targeting the periventricular nodules and MCD. Stereotactic EEG showed broadly distributed inter-ictal discharges in bilateral posterior hemispheres, with all of the active interictal contacts located in the cortical structures near electrode entry rather than at the deeper tissue around the cortical malformations. A total of 19 seizures were captured with eight right sided onset, four left sided onset, and seven appearing bilateral. This included thirteen of his habitual seizures, seven originating from the right hemisphere and four originating from the left hemisphere as well as two in which laterality could not be determined. Similar to the inter-ictal findings, all seizure onsets were in the parietal-occipital-temporal junction on the cortical surface overlying the nodules. Interestingly, he had preserved awareness with right sided seizures and, however, had loss of contact in left sided seizures or broad propagation.

**FIGURE 3:**
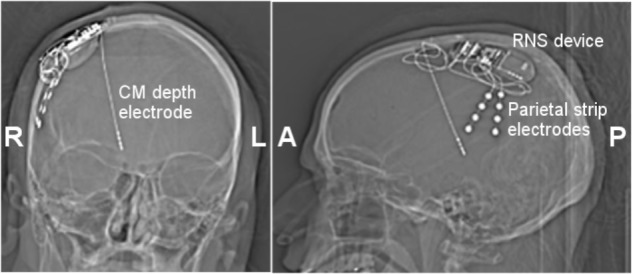
Coronal **(Left)** and lateral **(Right)** skull X-ray images. Three 4 contact electrodes were placed. A right centromedian depth electrode (1.5 mm contact size, 1.5 mm inter-contact spacing) and two parietal strip electrodes. One parietal strip electrode was connected to the RNS device, while the second was left unconnected. The RNS device was placed in an appropriately sized right craniectomy. CM, centromedian thalamus.

**FIGURE 4:**
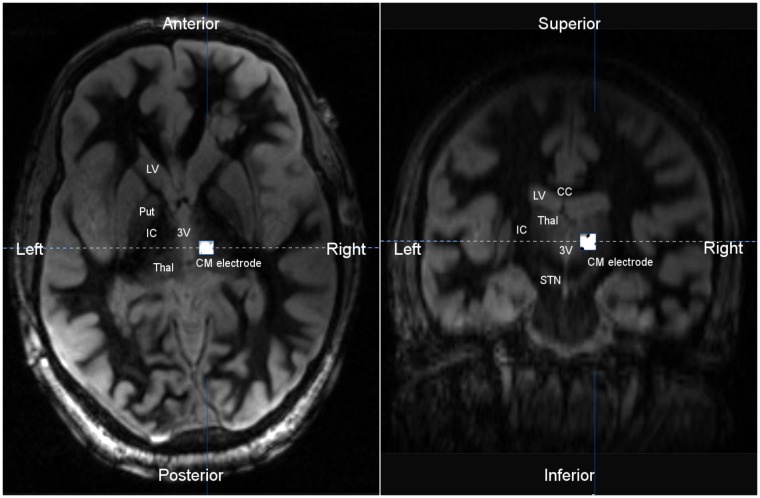
Axial **(Left)** and coronal **(Right)** T2 MR image co-registered with post-operative CT scan. The implanted centromedian (CM) depth electrode was localized to the central thalamus. The location of the depth electrode was verified by co-registration of a post-operative CT scan, with CM depth electrode artifact, and a pre-operative MRI. T2 imaging shows the thalamus bordered by the third ventricle (3V) and internal capsule (IC). LV, lateral ventricle; Put, putamen; Thal, thalamus; IC, internal capsule; CM, centromedian thalamus; STN, substantia nigra; CC, corpus callosum.

Surgical options including resection, laser ablation or RNS were considered. Surgical resection was not deemed a good option given the bilateral, broad posterior regions of onset with rapid spread. Similarly, laser ablation was not considered a good option. Ultimately there was consensus at the epilepsy surgery conference for centromedian (CM) thalamic RNS. An important factor in the decision was the broad connectivity to posterior association cortices as well as prior feasibility shown with centromedian DBS in patients suffering refractory epilepsy (discussed above). A depth electrode targeting the right CM thalamic nuclei was placed under MR image-guidance (ClearPoint, MRI Interventions; Irvine, CA, United States). The patient was then repositioned and using BrainLab stereotactic guidance (BrainLab; Munich, Germany), a craniectomy was performed near the right sided onset region. Two right parietal cortical strips were placed over the seizure onset zone for seizure detection as this was the most common site of seizure onset during the intracranial study. The RNS device (NeuroPace; Mountain View, CA, United States) (**Figure 3**) was implanted into the craniectomy site and the electrodes were connected; using the CM depth electrode and the most active of the two parietal strips. To assess adequate localization of the depth electrode, a thin-cut CT was performed and co-registered with the pre-op MRI. This technique is commonly utilized for localization of electrode contacts, using the CT for contact localization and MRI for good resolution within the thalamus (as seen in **Figure 4**).

**FIGURE 5:**
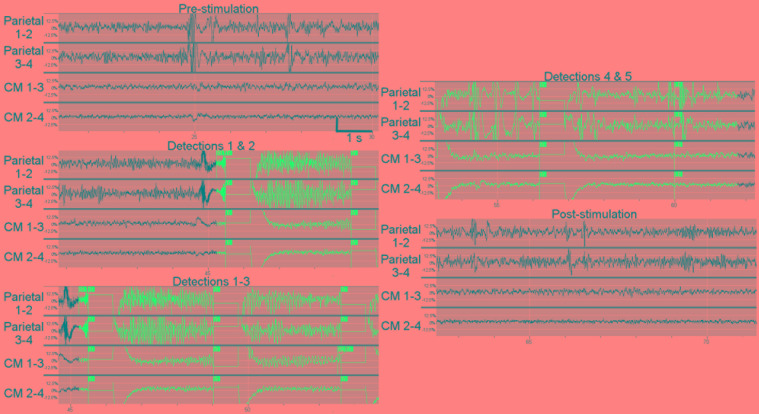
Example of *in vivo* ambulatory triggered stimulations in centromedian thalamus using responsive neurostimulation. This example shows triggered 1 s stimulations during an electrographic seizure or “long episode” seen on both the parietal electrodes and the centromedian thalamic electrodes. A long episode (LE marked in panel 5) is defined by five consecutive detections. At baseline (pre-stimulation), the high-frequency, high-amplitude activity in the parietal electrode contacts was distinct from the high-frequency low-amplitude background in the centromedian electrode contacts, however, some EEG spikes (such as when the amplifier for the parietal leads were saturated) were also noted in the CM. The first trigger was based on parietal electrode contacts 1–2 (labeled as detector B1 in panels 2 and 3), resulting in stimulation of CM electrode contacts; the seizure is clearly present in the parietal electrode contacts and also is visible in CM electrode contacts, particularly as the seizure builds as shown in the panel 3 containing detections 1–3. The manifestation of the seizure in CM results in detection and trigger based on the CM 1–3 electrode contacts (detection 3 in panel 3 which is labeled with “B2”). Detections 3–5 result from the seizure activity on the CM electrodes. After the fifth detection (labeled “LE”) the detector and stimulation are disabled. The seizure pattern fades in the CM thalamic electrodes. Notably, the parietal electrode contacts continued to display a high-frequency high-voltage signal.

As observed in prior studies (Velasco et al., 1987, 2006; Fisher et al., 1992; Velasco M. et al., 2000; Andrade et al., 2006; Valentin et al., 2013), we found that image-guided implantation resulted in accurate and safe implantation to the CM thalamus. Responsive stimulation in CM during seizures or false-triggers did not result in adverse side effects, changes in level of consciousness, or inadvertent perceptions (i.e., motor twitching, sensory changes, visual perceptions). This patient’s stimulation paradigm is two 40 Hz biphasic bursts lasting 200 ms (80 μs pulse width) of 1.5 mA current; this stimulation may be repeated up to five times based on persistence of the detection. Three of the four available detectors were used to trigger potential seizures: a line-length trigger in the parietal electrode; a bandpass filter 30–125 Hz in the parietal electrode with specified amplitude and duration thresholds; and an AUC measure in the centromedian electrode. Interestingly, as reported in prior CM DBS, stimulation of the deepest contact in our patient resulted in paresthesias, as expected based on the anatomically adjacent fibers of the medial lemniscus carrying sensory information. As there are no data reported from simultaneous cortical and CM thalamus recordings, the most pressing question for our group was whether intracranial recordings from the human CM thalamus could provide seizure related information or confirmation of network participation. In this initial report of ambulatory icEEG recordings from the human CM thalamus, the most notable observation is that many of the seizures as detected on the parietal strip are indeed present in the EEG from the CM electrode (**Figure 5**). We were pleasantly surprised at the ability of the CM electrode to capture seizures. In fact, we are now using the thalamic electrode as one of the detection sources as well as the stimulation target. During obvious seizures noted on the parietal strip, increasing amplitude in the 8–14 Hz range is noted in the CM EEG. Large cortical slow waves are also reflected in the CM electrode. Representation of seizure patterns in CM supports our hypothesis that the expected connectivity between these two nodes in the network play a role in seizures and may provide for neuromodulation.

In addition, it has been noted that the EEG from the parietal strip meets criteria for seizure most of the time, while simultaneous scalp EEG does not show any electrographic seizure. This finding confirms what we have seen in other intracranial studies and animal models, that small areas of underlying cortex can experience seizure or even be constantly in sub-clinical status epilepticus without alteration of the background scalp EEG. With the use of microwires, some have reported seizures on microwires without any background changes on neighboring macro-contacts (Stead et al., 2010). The CM thalamic electrode has episodes of electrographic seizures that appear to correlate more closely with the patient’s clinical seizures. We postulate that seizure propagation to and through the CM nucleus may play a role in clinical presentation of the seizure. With all detections, the CM nucleus receives a short burst of high-frequency stimulation (**Figure 5**). No data is yet available on the long-term effects of chronic CM responsive stimulation to seizure frequency, nor can we make any statements regarding clinical efficacy with a single patient.

Ongoing analysis is quantitating the effect of CM stimulation on resultant CM EEG characteristics, parietal strip electrode EEG characteristics, and patient-reported seizure outcomes. Long term intracranial monitoring paired with responsive stimulation over the course of months to years is essential to begin to understand the dynamic nature of the seizure circuits *in vivo*. Targeted placement of electrodes in central nodes of SGN, such as in the thalamus, is a next step in characterizing and modulating this seizure network in real-time.

## Future Directions

### Limitations of Responsive Neurostimulation

It takes considerable time, effort, and cost to develop and commercialize a brain implantable neurostimulation device for the control of seizures, and this limits the pace of innovation in this space. The NeuroPace RNS device is both revolutionary and limited in light of the requirements for controlling an epilepsy network. The RNS device technology is now more than a decade old. It can sample only a limited number of locations. This does not allow comprehensive sampling of a complete network. Nonetheless we have presented an argument in this manuscript that it has enough flexibility in how it can be implanted and programmed for the control of seizures in an SGN by targeting two primary nodes of the thalamo-cortical network. Furthermore, the RNS intervention is limited to stimulation in response to a detection. This does not allow more complex treatment based on network considerations both in terms of detection, and in terms of intervention. The detections are also limited to measures of activity at a location. Measures such as line length, bandpass power, and AUC are surrogate measures of excitability which reflect the concepts of an older conceptual understanding of seizure generation. These do not include measurements of network behavior such as synchrony or phase coupling. The intervention is performed through electrical stimulation. Electrical stimulation is limited to situations where a cathode and anode relationship can be formed. In the brain this is limited to two points which are close to each other. There is no provision for programming therapy for more complex considerations for example, where a node or a pair of nodes have to be controlled, and the control is contingent on an observed relationship between the nodes or a more complex set of network conditions.

### The Requirements for a BCI for the Control of Seizures

If seizures arise from a network and the components of this network must be monitored and controlled, an improvement in the capability of the BCI over the devices currently in use or under evaluation is suggested (Spencer et al., 2018). First, there is a need for a sensor network which can be flexibly placed to sample and monitor multiple nodes of a network. There may be multiple nodes in the network and the nodes may be neocortical or subcortical. The BCI will need an ability to use multi-contact subdural grid, strip and depth electrodes with a variety of configurations that allow for electrode arrays tailored for specific nodes or pathways. Second, as indicated above we will need to monitor for extensive periods of time to fully understand the network dynamics at play. This may preclude monitoring in the EMU due to the cost and limitations of extended inpatient monitoring and may require monitoring at home, possibly with an interface to wirelessly stream intracranial recordings and store data for extended periods of time. Third, we cannot assume that all nodes and pathways of the network will manifest aberrance in the same manner, or that this aberrance can be detected with the icEEG. Next generation BCI devices need multiscale and multimodal sensors to measure the icEEG and single unit/multi-unit electrophysiology, and sensors to measure additional modalities including neurochemistry. Fourth, the design of current devices is built on the previous conceptual understanding of seizure generation, and seeks to measure and control brain excitability. The need, however, is for evaluation of both brain activity and relationship. This will require the implementation of real-time network measures. Fifth, current approaches seek to control brain activity through electrical stimulation. Although electrical stimulation has proved to be a useful method of modulation, it is limited to the situation where two contacts are adjacent to each other and cannot be used over longer distances and for more complex geometries. There is a need to expand the intervention to modalities such as local drug delivery and focal brain cooling which can be used both at a point and over an area or volume. Sixth, the monitoring and intervention logic is currently limited to the monitoring and control of activity at a network node. There is a need for intervention logic to be defined not only for the monitoring and control of a node, but for networks composed of multiple nodes and pathways. The technology required to more fully monitor and control epilepsy networks exists, but needs to be integrated within a future BCI for the control of seizures.

## Ethics Statement

The present manuscript includes case report clinical, imaging and neurophysiological data from the RNS device. The subject presented in the case report gave written informed consent in accordance with the Declaration of Helsinki authorizing the use of clinical, imaging and RNS data for research and case report purposes. In addition, the RNS data is collected as part of a long term study of RNS data. This study is carried in accordance with the recommendations of the Yale Human Research Protection Program and the Yale University Institutional Review Board. The protocol was approved by the Yale University Institutional Review Board (HIC# 2000020827).

## Author Contributions

JG, HZ, and DS conceived and developed the theoretical framework of the manuscript. JG and DS designed and performed the surgical procedure of the case presentation. AG, JG, and HZ performed the data evaluation, analysis, and interpretation, designed the figures, designed and wrote the manuscript in consultation with DS with AG taking the lead in writing the manuscript. All of the authors participated in editing process with JG supervising the project.

## Conflict of Interest Statement

JG reports honorarium as a consultant for Medtronic, Inc. DS reports Honoraria as a member of the scientific review board for Monteris Medical, Inc. HZ and AG have no relevant conflicts of interest to report. None of these reported conflicts of interest are in relation to the work reported in the present manuscript.
